# Inhibition of RhoA/Rho kinase pathway and smooth muscle contraction by hydrogen sulfide

**DOI:** 10.1002/prp2.343

**Published:** 2017-09-04

**Authors:** Ancy D. Nalli, Hongxia Wang, Sayak Bhattacharya, Bryan A. Blakeney, Karnam S. Murthy

**Affiliations:** ^1^ Department of Physiology and Biophysics VCU Program in Enteric Neuromuscular Sciences Virginia Commonwealth University Richmond Virginia

**Keywords:** Cystathionine‐γ‐lyase, l‐cysteine, MLC_20_ phosphorylation, RhoA activity, signaling, sulfhydration

## Abstract

Hydrogen sulfide (H_2_S) plays an important role in smooth muscle relaxation. Here, we investigated the expression of enzymes in H_2_S synthesis and the mechanism regulating colonic smooth muscle function by H_2_S. Expression of cystathionine‐γ‐lyase (CSE), but not cystathionine‐β‐synthase (CBS), was identified in the colonic smooth muscle of rabbit, mouse, and human. Carbachol (CCh)‐induced contraction in rabbit muscle strips and isolated muscle cells was inhibited by l‐cysteine (substrate of CSE) and NaHS (an exogenous H_2_S donor) in a concentration‐dependent fashion. H_2_S induced S‐sulfhydration of RhoA that was associated with inhibition of RhoA activity. CCh‐induced Rho kinase activity also was inhibited by l‐cysteine and NaHS in a concentration‐dependent fashion. Inhibition of CCh‐induced contraction by l‐cysteine was blocked by the CSE inhibitor, dl‐propargylglycine (DL‐PPG) in dispersed muscle cells. Inhibition of CCh‐induced Rho kinase activity by l‐cysteine was blocked by CSE siRNA in cultured cells and DL‐PPG in dispersed muscle cells. Stimulation of Rho kinase activity and muscle contraction in response to CCh was also inhibited by l‐cysteine or NaHS in colonic muscle cells from mouse and human. Collectively, our studies identified the expression of CSE in colonic smooth muscle and determined that sulfhydration of RhoA by H_2_S leads to inhibition of RhoA and Rho kinase activities and muscle contraction. The mechanism identified may provide novel therapeutic approaches to mitigate gastrointestinal motility disorders.

**Abbreviations**
CBS: cystathionine‐β‐synthase
CCh: carbachol
CSE: cystathionine‐γ‐lyase
DL‐PPG: dl‐propargylglycine
MLCK: myosin light‐chain kinase
MLCP: myosin light‐chain phosphatase
NaHS: sodium hydrosulfide

## Introduction

Hydrogen sulfide (H_2_S) is produced via both enzymatic and nonenzymatic pathways in mammalian cells, but most of the H_2_S levels in tissues are attributed to enzymatic synthesis (Abe and Kimura 1996; Kamoun 2004; Caliendo et al. 2010; Wang 2012). The main enzymes responsible for H_2_S generation, cystathionine‐β‐synthase (CBS) and cystathionine‐γ‐lyase (CSE), are expressed in several tissues, but the pattern of expression has been reported to be tissue‐specific. Expression of CBS is mainly found in the central and peripheral nervous system, whereas expression of CSE is mainly found in the vascular system and liver (Bao et al. 1998; Renga 2011; Wang 2012). The H_2_S levels in the gastrointestinal (GI) system include two sources: a luminal sulfate‐reducing bacterial source in the large intestine and an endogenous generation by different cells within the wall of GI tract (Linden et al. 2010; Farrugia and Szurszewski 2014). Regulation of GI functions including motility and secretion by H_2_S has been reported previously (Distrutti et al. 2006; Linden et al. 2008; Hennig and Diener 2009; Xu et al. 2009; Wallace 2010; Strege et al. 2011; Sha et al. 2014; Nalli et al. 2015).

Relaxation of vascular muscle in response to H_2_S was documented in the aorta, portal vein, and mesenteric artery (Wang 2012). H_2_S also inhibits the motility in different regions of the GI tract in different species. In the GI tract, studies using whole segments of the intestine or muscle strips from rat, mouse, guinea pig, and human have shown that H_2_S inhibits contraction (Dhaese and Lefebvre 2009; Gil et al. 2011, 2013; Kasparek et al. 2012; Martinez‐Cutillasa et al. 2015). The spontaneous contractions in colonic muscle strips from mouse, rat, and human were inhibited by NaHS (Gallego et al. 2008). Inhibition of spontaneous contractions by NaHS was not affected by the neural blocker, tetrodotoxin, but significantly reduced by K_ATP_ channel blocker, glibenclamide, and apamin suggesting that the effect of NaHS was independent of neural activation and probably involved activation of K_ATP_ channels as well as apamin‐sensitive small conductance K^+^ channels (Gallego et al. 2008). In circular muscle of rat ileum, but not longitudinal muscle, the effect of NaHS was partly inhibited by glibenclamide (Nagao et al. 2011, 2012). In longitudinal muscle of rat jejunum, the inhibitory effect of NaHS on stimulated contractile activity was blocked by glibenclamide without affecting the spontaneous contractile activity (Kasparek et al. 2012). In contrast, studies by Teague et al. (2002) in rabbit and guinea pig ileum demonstrated that NaHS caused inhibition of acetylcholine‐ or electrical field‐stimulated muscle contraction and the effect was independent of K_ATP_ channel activation as glibenclamide had no effect on NaHS‐induced decrease in muscle contraction.

Expression of CSE and/or CBS has been reported in different cell types in the GI tract including enteric neurons, interstitial cells of Cajal (ICC), and epithelial cells (Schicho et al. 2006; Martin et al. 2010; Kasparek et al. 2012; Sha et al. 2014; Quan et al. 2015). Hence, the effect of H_2_S could be due to release of neurotransmitters from the enteric neurons, inhibition of pacemaker activity of ICC, and/or its direct inhibitory effect on smooth muscle function. Although the evidence for the enzymatic production of H_2_S by GI tissues was produced, the evidence for the regulation of synthesis is not clear. The rate of H_2_S production in intact mouse colonic tissue in the presence of 10 mmol/L cysteine in Kreb's solution was shown to be around 0.6 pmol/min/mg (Linden et al. 2008, 2010). In the small and large intestine of rat, the basal level of H_2_S was reported to be around 60 nmol/g/h and the response to 10 mmol/L cysteine in these tissues ranged from 300 to 500 nmol/g/h, a 5‐ to 10‐fold increase (Martin et al. 2010).

Phosphorylation of serine 19 on the 20‐kDa regulatory light‐chain of myosin II (MLC_20_) is an essential step in smooth muscle contraction (Hartshorne et al. 1998; Somlyo and Somlyo 2003; Murthy 2006; de Godoy and Rattan 2011). Phosphorylation of MLC_20_ is regulated by a Ca^2+^/calmodulin‐dependent MLC kinase (MLCK), which initiates phosphorylation of MLC_20_ and MLC phosphatase (MLCP), which dephosphorylates MLC_20_. MLCP is a heterotrimer with an 110‐ to 130‐kDa regulatory subunit (myosin phosphatase target subunit 1 (MYPT1), a 37‐kDa catalytic subunit of type 1 phosphatase (PP1cδ), and a 20‐kDa subunit of unknown function (Hartshorne et al. 1998).

The RhoA/Rho kinase signaling pathway regulates muscle contraction. RhoA is a small G protein with inherent GTPase activity and cycles between two states: an inactive GDP‐bound state and an active GTP‐bound state. Activated RhoA binds to the Rho‐binding domain of Rho kinase, causing the enzyme to unfold and freeing its catalytic activity. Phosphorylation of MYPT1 at Thr^696^ by Rho kinase causes its dissociation from, and inhibition of, the catalytic subunit resulting in MLC_20_ phosphorylation and muscle contraction (Hartshorne et al. 1998; Somlyo and Somlyo 2003; Murthy 2006; de Godoy and Rattan 2011).

Our results demonstrate selective expression of CSE in colonic smooth muscle cells of rabbit, mouse, and human, and addition of l‐cysteine or NaHS causes S‐sulfhydration of RhoA that lead to inhibition of RhoA and Rho kinase activities and muscle contraction in response to contractile agonists.

## Materials and Methods

### Reagents

*C*ystathionine‐β‐synthase (CBS) and cystathionine‐γ‐lyase (CSE) antibodies were purchased from Proteintech (Chicago, IL); [^32^P]ATP was purchased from PerkinElmer (Cambridge, MA); HRP‐conjugated secondary antibodies were obtained from Cell Signaling Technology (Danvers, MA); PVDF membranes were obtained from Millipore (Billerica, MA); Effectene Transfection Reagent, QIAEX^®^II was from Qiagen (Germantown, MD); culture medium (Dulbecco's modified Eagle's medium) was from Fisher Scientific (Ashville, NC); l‐Cysteine and dl‐propargylglycine (PPG) were from Sigma (St. Louis, MO). All other reagents were from Sigma (St. Louis, MO).

Rabbits (New Zealand white male) weighing 4–5 lbs were purchased from RSI Biotechnology (Clemmons, NC), and mice (male C57BL/6 strain) were purchased from Jackson Laboratories (Bar Harbor, ME). Rabbits and mice were acclimatized at the facility administered by the Division of Animal Resources, Virginia Commonwealth University. The Institutional Animal Care and Use Committee of Virginia Commonwealth University approved all the procedures conducted. Colons from normal human subjects were obtained from a nonprofit organization known as National Disease Research Interchange (NDRI, Philadelphia, PA) that provides human organs and tissue. The studies involving human tissues are approved as exempt from VCU Institutional Review Board.

### Isolation of smooth muscle cells

The colon from rabbit, mouse, and human were dissected out and after emptying the contents was placed in oxygenated Kreb's solution composed of 118 mmol/L NaCl, 4.75 mmol/L KCl, 1.19 mmol/L KH_2_PO_4_, 1.2 mmol/L MgSO_4_, 2.54 mmol/L CaCl_2_, 25 mmol/L NaHCO_3_, 11 mmol/L glucose at 37°C, and pH 7.4. Small colonic segments were threaded onto a glass rod. The longitudinal muscle with adherent myenteric plexus was removed by radial abrasion with Kim wipes. Circular muscle layer of the colon was used to isolate smooth muscle cells as described previously (Murthy et al. 2003a,b; Rajagopal et al. 2013; Nalli et al. 2015). Briefly, colonic circular muscle strips were incubated for 30 min at 31°C in 15 mL of HEPES medium (120 mmol/L NaCl, 4 mmol/L KCl, 2.6 mmol/L KH_2_PO_4_, 0.6 mmol/L MgCl_2_, 25 mmol/L HEPES, 14 mmol/L glucose, 2.1% (v/v) Eagle's essential amino acid mixture, 0.1% collagenase [type II], and 0.1% soybean trypsin inhibitor). After the 30‐min digestion period, tissues were washed with collagenase‐free medium (50 mL) and muscle cells were allowed to disperse spontaneously. Cells were collected by filtration through 500 μm Nitex followed by centrifugation twice at 350*g* for 10 min. Smooth muscle cells were cultured in DMEM containing 10% fetal bovine serum and cells passaged once after attaining confluence. For experiments, muscle cells were used in the first passage.

### Transfection of CSE siRNA

The eukaryotic expression vector pcDNA3 was used to subclone CSE siRNA into the multiple cloning sites (*EcoRI*). Smooth muscle cells in culture were transiently transfected for 48 h with recombinant plasmid cDNAs. To monitor transfection efficiency, muscle cells were cotransfected with 2 μg pcDNA3 vector and 1 μg of pGreen Lantern‐1 DNA (Rajagopal et al. 2013; Nalli et al. 2015).

### Western blot analysis

Expression of CSE and CBS was measured by western blot as described previously (Murthy et al. 2003a,b; Rajagopal et al. 2013; Nalli et al. 2015). Smooth muscle cells were homogenized in lysis buffer containing Triton X‐100 and protease and phosphatase inhibitors. The lysates were centrifuged at 20,000*g* for 10 min at 4°C and the supernatants were collected. Aliquots containing an equal amount of protein (50 μg) were resolved on 10% SDS‐PAGE and the proteins were transferred to PVDF membranes. The membranes were incubated overnight with CSE or CBS antibodies followed by incubation with appropriate secondary antibody conjugated with horseradish peroxidase. Enhanced chemiluminescence was used to visualize protein bands on the membrane.

### Assay for RhoA GTPase activation

RhoA activation was evaluated in pull‐down assays using specific anti‐RhoA‐GTP antibody and protein A/G beads (Rajagopal et al. 2013). In brief, cultured smooth muscle cells were incubated with carbachol (CCh, 1 μmol/L) for 10 min. In some experiments, CCh was added after treatment with l‐cysteine (10 mmol/L) or NaHS (1 mmol/L) for 10 min. In some cases, the cells were preincubated with CSE inhibitor, DL‐PPG (1 mmol/L) for 10 min before the addition of CCh in the presence or absence of l‐cysteine or NaHS. Cells were homogenized in the lysis buffer and GTP‐bound RhoA was immunoprecipitated using monoclonal antibody (NewEast Biosciences, Malvern, PA) that specifically recognizes RhoA‐GTP. RhoA‐GTP bound antibody were pulled down by protein A/G, washed with lysis buffer, and processed for separation by SDS‐PAGE. The level of activated RhoA was evaluated by western blot analysis.

### Biotin switch assay to detect S‐sulfhydration

S‐sulfhydration was measured by biotin switch assay as described previously (Jaffrey and Snyder 2001; Mustafa et al. 2009; Kang et al. 2015) with modification. HEK293 cells transfected with RhoA cloned pcDNA 3 vector were homogenized in a medium A (250 mmol/L HEPES‐NaOH [pH 7.7], 1 mmol/L EDTA, 2.5% SDS, 0.1 mmol/L neocuproine) containing 100 μmol/L deferoxamine. Protein samples (250 μg) were then treated in the presence or absence of NaHS (0.1 mmol/L and 1 mmol/L) for 15 min and then incubated at 50°C for 20 min with blocking buffer (medium A adjusted to 2.5% SDS and 20 mmol/L methyl methane thiosulfonate) with frequent vortexing. Proteins were precipitated using acetone and incubated at 37°C for 3 h in medium A adjusted to 1% SDS with 4 mmol/L biotin‐HPDP (N‐[6‐(biotinamido) hexyl]‐3′‐(2′‐pyridyldithio)‐propionamide) in dimethyl formamide. The biotinylated proteins were precipitated by streptavidin‐agarose beads, washed with medium A, and resolved by SDS‐PAGE. After the transfer of proteins onto PVDF membranes, RhoA was analyzed by western blot.

### Assay for Rho kinase activity

Rho kinase activity was measured by immunokinase assay as previously described (Murthy et al. 2003a,b; Rajagopal et al. 2013; Nalli et al. 2015). Smooth muscle cells were treated with different concentrations of NaHS or l‐cysteine for 10 min and then with CCh for 10 min. The cells were homogenized in medium containing 50 mmol/L Tris‐HCl (pH 7.5), 0.1% SDS, 0.5% sodium deoxycholate, 1% Nonidet P‐40, 150 mmol/L NaCl, 1 mmol/L PMSF, 10 μg/mL aprotinin, 10 μg/mL pepstatin A, and 10 μg/mL leupeptin. Aliquots containing an equal amount of protein (50 μg) were incubated overnight at 4°C with Rho kinase‐2 antibody and protein A/G agarose. The immunoprecipitates collected by centrifugation were washed twice with a medium containing 10 mmol/L MgCl_2_ and 40 mmol/L HEPES (pH 7.4) followed by incubation for 5 min at 4°C with myelin basic protein (MBP) (1 mg/mL). The reaction was initiated by the addition of 10 μCi of [^32^P]ATP (3000 Ci/mmol) and 20 μmol/L ATP at 37°C and terminated after 10 min by spotting the reaction mixture onto phosphocellulose disks to capture phosphorylated MBP. The disks were washed with 75 mmol/L phosphoric acid to remove free radioactivity. Phosphorylation of MBP was determined from the radioactivity on disks by liquid scintillation.

### Measurement of contraction in muscle strips

Rabbit colonic muscle strips cut in the direction of circular muscle layer from rabbit colon were rinsed immediately in Kreb's medium containing 118 mmol/L NaCl, 4.8 mmol/L KCl, 1 mmol/L MgSO_4_, 1.15 mmol/L NaH_2_PO_4_, 15 mmol/L NaHCO_3_, 10.5 mmol/L glucose, and 2.5 mmol/L CaCl_2_. Strips with the aid of silk threads were suspended vertically in 5 mL tissue bath containing oxygenated (95% O_2_/5% CO_2_) Kreb's medium (pH of 7.4) at 37°C. The isometric force generated by circular muscle was measured by mounting the tissue between a glass rod and isometric transducer (Grass Technologies, Quincy MA) connected to a computer recording system. A resting tension of 1 g was given and the muscle strips were allowed to equilibrate. Muscle strips were contracted with 10 μmol/L CCh in the presence or absence of different concentrations of l‐cysteine or NaHS pretreatment for 10 min. In some experiments, muscle strips were pretreated with 10 μmol/L glibenclamide (a K_ATP_ channel blocker) before the addition of NaHS or l‐cysteine. Tissue weight was recorded after the experiment and contraction was calculated as area under the curve in response to CCh alone and compared with CCh with l‐cysteine or NaHS (Nalli et al. 2015).

### Measurement of contraction in muscle cells

Contraction in dispersed muscle cells was determined by scanning micrometry as described previously (Murthy et al. 2003a,b; Rajagopal et al. 2013; Nalli et al. 2015). An aliquot of muscle cells (0.4 mL containing 10^4^/cell mL) was preincubated with a different concentration of l‐cysteine or NaHS for 10 min and then with CCh for another for 10 min. A final concentration of 1% acrolein was used to terminate the reaction and a drop of cell suspension was placed on a slide under a cover slip. In some experiments, muscle cells were pretreated with 10 μmol/L glibenclamide (a K_ATP_ channel blocker) before the addition of NaHS or l‐cysteine. Cell length was measured by scanning micrometry. Cell length in the absence of any treatment was taken as resting cell length. Contraction was expressed as a decrease in mean cell length from control cell length.

### Statistical analysis

Data are expressed as mean ± SEM. “*n*” represents average values of one sample run in duplicate or triplicate from one animal. Comparisons between groups were analyzed by Student's *t*‐test. A *P* < 0.05 was considered statistically significant. Statistical analyses were performed using the Prism software program (GraphPad Software, San Diego, CA).

## Results

### Expression of CSE protein in smooth muscle cells

**Figure 1:**
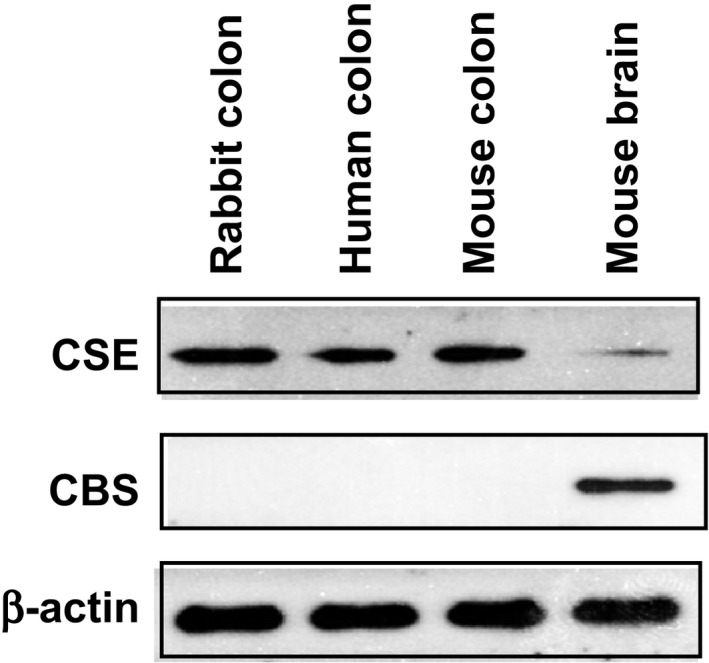
Expression of cystathionine‐γ‐lyase (CSE) in colonic smooth muscle. Smooth muscle cells were isolated from the colon of rabbit, mouse, and human, and cultured in DMEM‐10. Lysates were prepared from cultured muscle cells and expression of CSE and CBS were analyzed by western blot. Expression of CSE (66 kDa), but not CBS, was detected in colonic smooth muscle from rabbit, mouse, and human, and also in mouse brain. Expression of CBS (61 kDa) was detected in lysates derived from the mouse brain. Representative images from four separate experiments are shown in the figure.

Expression studies by western blot demonstrated the presence of CSE protein (66 kDa) in cultured muscle cells of the colon of mouse, rabbit, and human (Fig. 1). Under similar conditions, there was no detectable expression of CBS protein in these samples. However, expression of CBS protein was demonstrated using CBS‐specific antibody in mouse brain (Fig. 1). The presence of smooth muscle‐specific γ‐actin and absence of markers for interstitial cells of Cajal and endothelial cells determined the purity of these cultured smooth muscle cells in previous studies (Teng et al. 1998). Selective expression of CSE in smooth muscle cells is consistent with the tissue‐specific expression of CSE and CBS (Schicho et al. 2006; Linden et al. 2010; Wang 2012).

### Inhibition of contraction by H_2_S in muscle strips

Isometric contraction was measured in muscle strips isolated from the colon of rabbit. Muscle strips were equilibrated to a passive tension of 1 g for 1 h before experiments were conducted. Area under curve (AUC) measurement representing force versus time in response to CCh (10 μmol/L) was defined as muscle contraction above basal. AUC was recorded for the first 2 min in response to CCh. The strips were incubated with various concentrations of l‐cysteine (0.1 μmol/L to 10 mmol/L) or NaHS (0.01 μmol/L to 1 mmol/L) for 10 min before the addition of CCh and measurements of AUC were recorded again to estimate the amount of inhibition of contraction in response to l‐cysteine or NaHS.

**Figure 2:**
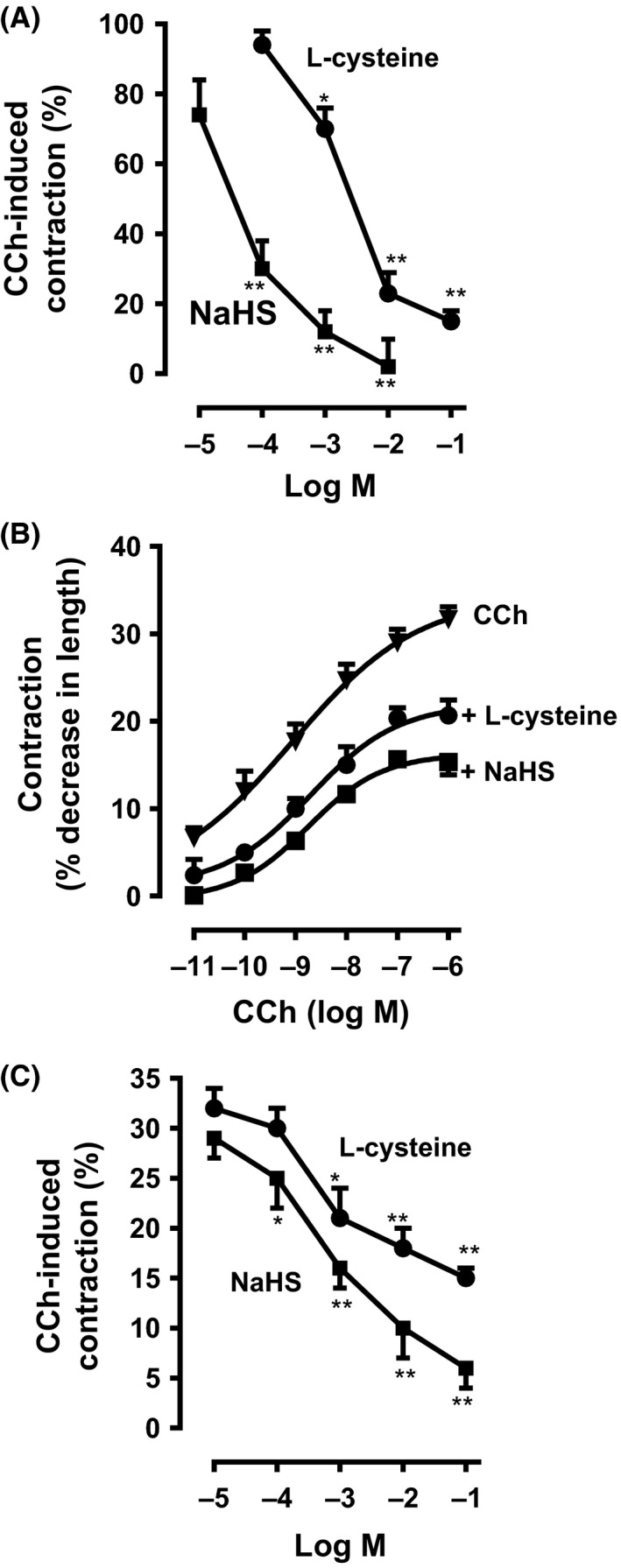
Inhibition of carbachol‐induced contraction by l‐cysteine and NaHS in colonic smooth muscle. (A) Muscle strips from the rabbit colon were allowed to equilibrate to a passive tension of 1 g for 1 h and then treated with carbachol (carbachol [CCh], 10 μmol/L). The increase in tension (area under the curve [AUC]) in response to CCh from *t* = 0 min to *t *= 2 min was calculated as a contraction. In separate experiments, muscle strips were incubated with different concentrations of l‐cysteine (0.1 to 100 mmol/L) or sodium hydrosulfide (NaHS, 0.01 to 10 mmol/L) for 10 min before addition of carbachol. Inhibition of contraction was measured as a percent decrease in AUC in the presence of l‐cysteine or NaHS compared to control response. CCh induced contraction of 369 ± 26 gram‐seconds (AUC) above basal tension. Values are mean ± SEM of four experiments. *P < 0.05; **P < 0.01, significant inhibition of CCh‐induced muscle contraction. (B) Muscle cells isolated from circular muscle layer of rabbit colon were treated with increasing concentrations of CCh (0.01 nmol/L to 1 μmol/L) for 10 min to induce sustained contraction. In some experiments, cells were pretreated with l‐cysteine (10 mmol/L) or NaHS (1 mmol/L) for 10 min and then treated with CCh for 10 min. Muscle cell length was measured by scanning micrometry. Contraction by CCh was calculated as the decrease in muscle cell length from basal cell length of 109 ± 5 μm. Values are mean ± SEM of five experiments. (C) Muscle cells isolated from rabbit colon were treated with CCh (0.1 μmol/L) for 10 min to induce sustained contraction. In some experiments, cells were pretreated with different concentrations of l‐cysteine (0.01 mmol/L to 100 mmol/L) or NaHS (0.01 mmol/L to 100 mmol/L) for 10 min and then treated with CCh for 10 min. Muscle cell length was measured by scanning micrometry. Contraction by CCh was calculated as a percent decrease in muscle cell length from basal cell length (30 ± 3% decrease in cell length from the basal cell length of 109 ± 5 μm). Values are mean ± SEM of seven experiments. *P < 0.05; **P < 0.01, significant inhibition of CCh‐induced muscle contraction.

As shown previously (Nalli et al. 2015), CCh (10 μmol/L) induced a contraction of 367 ± 26 gram‐seconds above basal tension (*n* = 4). CCh‐induced contraction was inhibited by l‐cysteine and NaHS in a concentration‐dependent fashion (Fig. 2A). In vascular and visceral smooth muscle, the inhibitory effect of H_2_S was shown to be mediated by activation of plasma membrane K_ATP_ channels and hyperpolarization (Zhao et al. 2001; Zhao and Wang 2002; Tang et al. 2005; Gallego et al. 2008; Mustafa et al. 2011; Wang 2012; Gade et al. 2013). Incubation of colonic muscle strips with glibenclamide (10 μmol/L) for 10 min did not affect the inhibitory effect of l‐cysteine (10 mmol/L) (65 ± 4% inhibition vs. 61 ± 3% inhibition in the presence of glibenclamide) or NaHS (1 mmol/L) (82 ± 6% inhibition vs. 79 ± 7% inhibition in the presence of glibenclamide). Glibenclamide had no effect on CCh‐induced contraction (329 ± 21 gram‐seconds above basal tension vs. 309 ± 35 gram‐second in the presence of glibenclamide, *n* = 3), suggesting that inhibition of CCh‐induced contraction by l‐cysteine was not due to activation of K_ATP_ channels.

### Inhibition of muscle contraction by H_2_S in isolated muscle cells

Contractile agonists (e.g., CCh) induce biphasic contraction in muscle cells: a rapid contraction within 30 sec followed by a sustained contraction for 20 min (Murthy et al. 2003a). The effect of l‐cysteine and NaHS on sustained contraction was measured. Muscle cells were pretreated with l‐cysteine (10 mmol/L) or NaHS (1 mmol/L) and then treated with different concentrations of CCh (0.01 nmol/L to 1 μmol/L) for 10 min. CCh caused a concentration‐dependent contraction in muscle cells with EC_50_ of 10^−9^ ± 3 × 10^−10^ mol/L. Maximal contraction (32 ± 4% decrease in cell length from control length of 109 ± 5 μm, *n* = 7) was obtained with 1 μmol/L of CCh. Addition of l‐cysteine or NaHS shifted the contractile response to CCh to the right with EC_50_ values of 3.5 × 10^−8^ ± 5 × 10^−9^ mol/L and 9 × 10^−8^ ± 7 × 10^−9^ mol/L, respectively, suggesting inhibition of CCh‐induced contraction by H_2_S (Fig. 2B).

Contraction in response to maximal dose of CCh (1 μmol/L) was inhibited by l‐cysteine or NaHS in a concentration‐dependent fashion (Fig. 2C). Glibenclamide, a K_ATP_ channels blocker, was used to test whether the effect of H_2_S involved activation of K_ATP_ channels. Pretreatment with glibenclamide (10 μmol/L) for 10 min did not affect the inhibition of contraction by l‐cysteine (10 mmol/L) (53 ± 2% inhibition alone vs. 52 ± 4% inhibition in the presence of glibenclamide, *n* = 6) or NaHS (1 mmol/L) (63 ± 3% inhibition alone vs. 60 ± 6% inhibition in the presence of glibenclamide, *n* = 6). Glibenclamide had no effect on CCh‐induced contraction (31 ± 3% contraction vs. 29 ± 2% contraction in the presence of glibenclamide, *n* = 4). These results demonstrate that H_2_S inhibits muscle contraction and the effect of H_2_S does not involve K_ATP_ channel activation. Control studies showed that inhibition of contraction by levcromakalim (10 μmol/L), a potassium channel activator, was reversed by glibenclamide (52 ± 7% inhibition of contraction alone vs. 8 ± 4% inhibition in the presence of glibenclamide).

**Figure 3:**
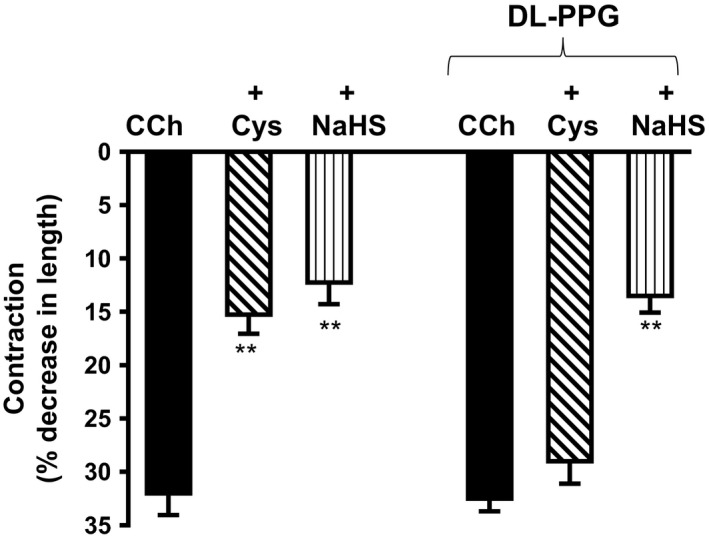
Effect of cystathionine‐γ‐lyase (CSE) inhibitor, dl‐propargylglycine (DL‐PPG) on l‐cysteine‐induced inhibition of sustained contraction. Muscle cells isolated from colon were treated with carbachol (CCh) (1 μmol/L) for 10 min in the presence or absence of l‐cysteine (10 mmol/L) or NaHS (1 mmol/L) pretreatment for 10 min. In some experiments, cells were pretreated with CSE inhibitor DL‐PPG (1 mmol/L) for 10 min before the addition l‐cysteine (10 mmol/L) or NaHS (1 mmol/L) for another 10 min. Muscle cell length was measured by scanning micrometry. Contraction in response to CCh was calculated as a percent decrease in muscle cell length from control cell length (32 ± 4% decrease in cell length from the basal cell length of 109 ± 6 μm). Values are mean ± SEM of four experiments. **P < 0.01, significant inhibition of CCh‐induced muscle contraction.

Inhibition of CCh‐induced contraction by l‐cysteine was blocked by treatment of cells with the CSE inhibitor, dl‐propargylglycine (DL‐PPG) (1 mmol/L) for 10 min (Fig. 3). Treatment of cells with l‐cysteine (10 mmol/L) for 10 min caused significant inhibition (59 ± 3% inhibition) of contraction in response to CCh. The inhibitory effect of l‐cysteine was attenuated (15 ± 8% inhibition) in the presence of DL‐PPG (1 mmol/L). In contrast, inhibition of sustained contraction by NaHS (1 mmol/L) was not affected by DL‐PPG (59 ± 3% inhibition vs. 60 ± 9% inhibition with DL‐PPG), suggesting that the inhibition of muscle contraction by l‐cysteine was mediated by the activation of CSE and generation of H_2_S.

**Figure 4:**
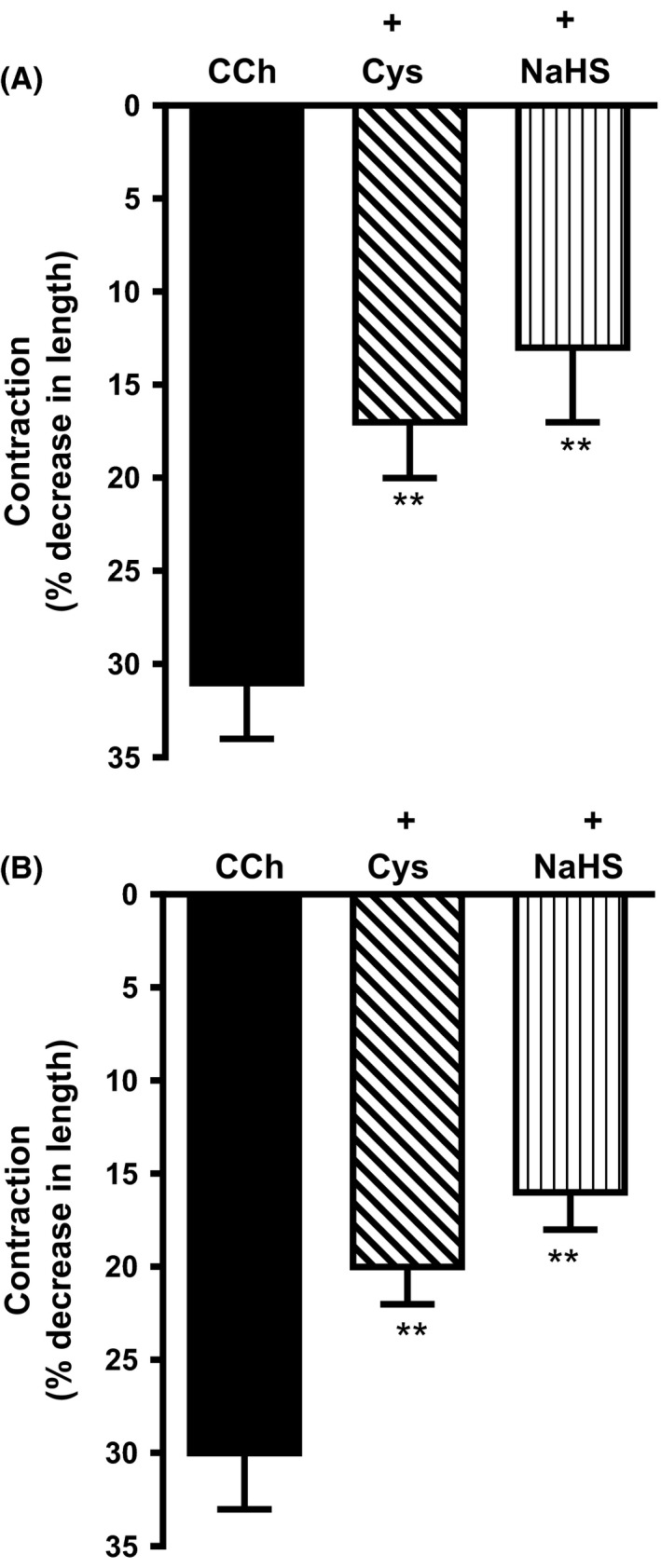
Inhibition of carbachol‐induced contraction by l‐cysteine and NaHS in colonic smooth muscle. Muscle cells isolated from circular muscle layer of the mouse (top panel) and human colon (bottom panel) were pretreated with l‐cysteine (10 mmol/L) or NaHS (1 mmol/L) for 10 min and then treated with carbachol (CCh; 1 μmol/L) for 10 min to induce sustained contraction. Muscle cell length was measured by scanning micrometry. Contraction by CCh was calculated as the decrease in muscle cell length from basal cell length (31 ± 3% decrease in cell length from the basal cell length of 115 ± 7 μm in mouse colon and 30 ± 3% decrease in cell length from the basal cell length of 93 ± 6 μm in the human colon). Values are mean ± SEM of 5–7 experiments. **P < 0.01, significant inhibition of CCh‐induced muscle contraction.

Pretreatment of cells with l‐cysteine (10 mmol/L) or NaHS (1 mmol/L) for 10 min also inhibited sustained contraction in mouse and human. In mouse colonic muscle cells, contraction (31 ± 3% decrease in cell length) in response to 1 μmol/L CCh was inhibited by l‐cysteine (45 ± 4% inhibition) and NaHS (57 ± 6% inhibition) (Fig. 4A). In human colonic smooth muscle cells, contraction (30 ± 3% decrease in cell length) in response to 1 μmol/L CCh was inhibited by l‐cysteine (34 ± 3% inhibition) and NaHS (47 ± 5% inhibition) (Fig. 4B).

### Inhibition of CCh‐induced RhoA activity by H_2_S

Previous studies in GI smooth muscle have shown that sustained MLC_20_ phosphorylation and contraction were mediated by RhoA/Rho kinase pathway (Murthy et al. 2003a), raising the possibility that inhibition of contraction by H_2_S could be due to inhibition of this pathway.

**Figure 5:**
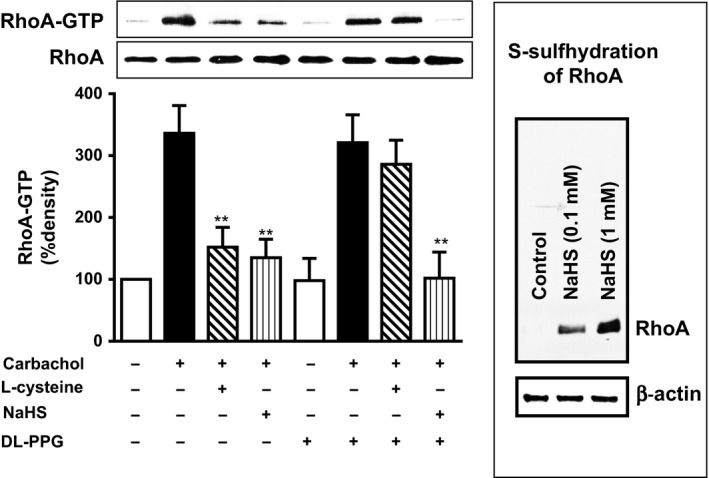
S‐Sulfhydration of RhoA and inhibition of carbachol‐induced RhoA activity by l‐cysteine and NaHS in colonic smooth muscle. (A) Muscle cells isolated from circular layer of rabbit colon were treated with carbachol (CCh) (1 μmol/L) 10 min in the presence or absence of the l‐cysteine (10 mmol/L) or NaHS (1 mmol/L) pretreatment for 10 min. In some experiments, cells were pretreated DL‐PPG (1 mmol/L) for 10 min before the addition of l‐cysteine or NaHS for another 10 min. RhoA activity in response to CCh was measured by incorporation of GTP into RhoA by western blot using an antibody that is specific for RhoA‐GTP. The image depicts representative blot of four separate experiments. Values are mean ± SEM of four experiments. **P < 0.01, significant inhibition of CCh‐induced RhoA activity. (B) Lysates obtained from HEK cells were treated in the presence or absence of NaHS (0.1 or 1 mmol/L) for 15 min and blocked with methyl methanethiosulfonate (MMTS). H_2_S modified ‐SSH residues are labeled with N‐[6‐(biotinamido) hexyl]‐3′‐(2′‐pyridyldithio)‐propionamide (HPDP‐biotin) and biotinylated proteins were pulled down and analyzed by western blot. Western blot analysis showed the difference in the sulfhydration levels in the control and NaHS‐treated samples. The figure depicts representative blot of three separate experiments.

RhoA is a small G protein that is bound to GDP in the basal state and GTP in the activated state. Addition of CCh (1 μmol/L) for 10 min caused an increase in the RhoA activity, measured as an increase in the incorporation of GTP into RhoA using an antibody that specifically recognizes RhoA‐GTP. Pretreatment of cells with l‐cysteine (10 mmol/L) or NaHS (1 mmol/L) for 10 min caused inhibition of RhoA activity in response to CCh (Fig. 5A). These results suggest that endogenous and exogenous H_2_S inhibits RhoA activity in colonic muscle cells. The notion that l‐cysteine exerts its effect via activation of CSE was examined using DL‐PPG. Pretreatment of cells with DL‐PPG (1 mmol/L) for 10 min reversed the effect of l‐cysteine on RhoA activity in response to CCh (Fig. 5A). DL‐PPG, in contrast, had no effect on NaHS‐induced inhibition of RhoA activity in response to CCh (Fig. 5A).

### S‐Sulfhydration of RhoA by H_2_S

S‐sulfhydration is known to be the primary mechanism through which H_2_S signals. H_2_S alters the function of K_ATP_ channels via S‐sulfhydration (Mustafa et al. 2009; Wang 2012). Therefore, we examined if H_2_S alters the activity of RhoA via S‐sulfhydration. S‐sulfhydration of RhoA in response to NaHS (0.1 and 1 mmol/L) was analyzed using a biotin switch assay. Basal sulfhydration of RhoA was not detected in control cells (Fig. 5B). Addition of NaHS for 15 min, however, caused sulfhydration of RhoA, suggesting that inhibition of RhoA activity by H_2_S could be due to S‐sulfhydration of RhoA (Fig. 5B).

### Inhibition of Rho kinase activity by H_2_S

**Figure 6:**
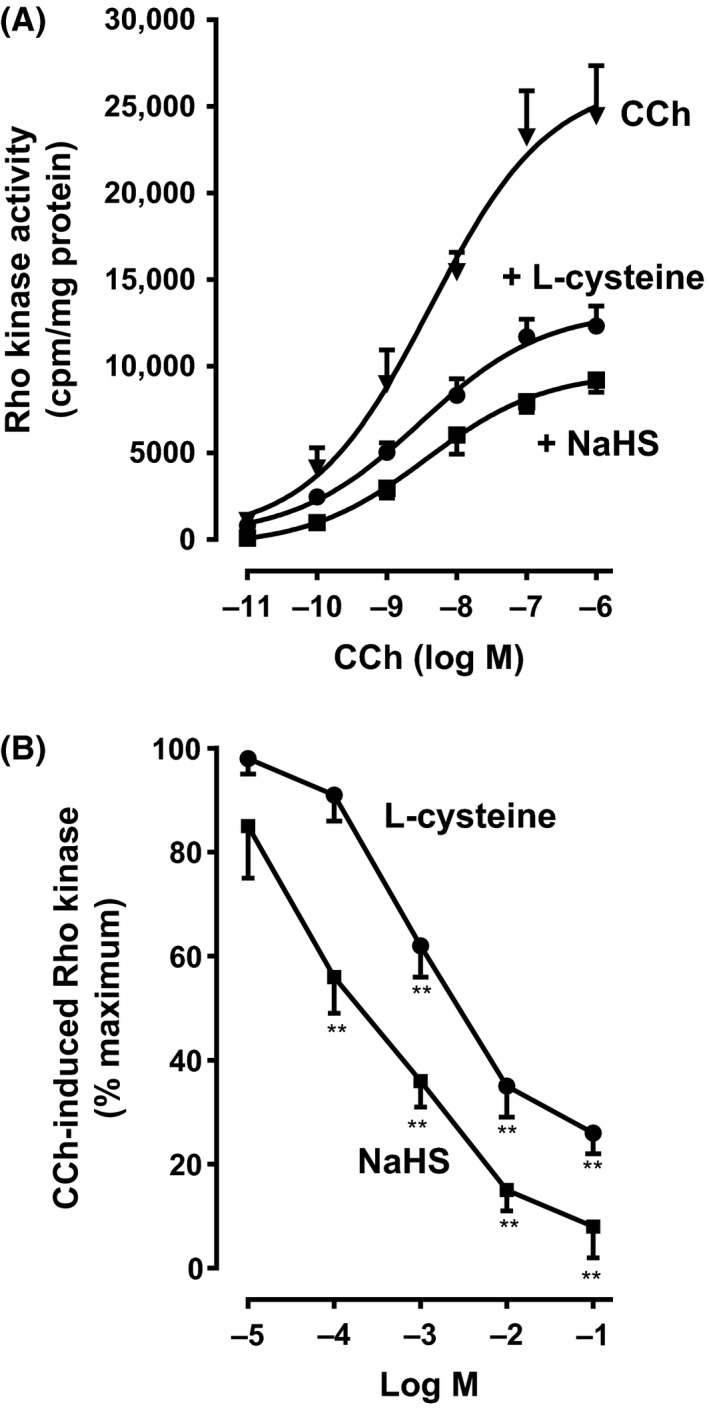
Inhibition of carbachol‐induced Rho kinase activity by l‐cysteine and NaHS in colonic muscle cells. (A) Muscle cells isolated from rabbit colon were treated with different concentrations of carbachol (CCh) for 10 min. In some experiments, cells were pretreated with l‐cysteine (10 mmol/L) or NaHS (1 mmol/L) for 10 min and then treated with CCh for 10 min. Rho kinase activity was measured by immunokinase assay using [^32^P]ATP. Results are expressed as cpm/mg protein. Values are mean ± SEM of 4–6 experiments. (B) Muscle cells isolated from rabbit colon were treated with CCh (1 μmol/L) for 10 min. In some experiments, cells were pretreated with different concentrations of l‐cysteine (0.01 mmol/L to 100 mmol/L) or NaHS (0.01 mmol/L to 100 mmol/L) for 10 min and then treated with CCh (1 μmol/L) for 10 min. Rho kinase activity was measured by immunokinase assay using [^32^P]ATP. Results are expressed as a percent inhibition of CCh‐induced Rho kinase activity. Values are mean ± SEM of 4–6 experiments. **P < 0.01, significant inhibition of CCh‐induced Rho kinase activity.

Contractile agonists induce an increase in Rho kinase activity in smooth muscle via activation of RhoA. Consistent with the previous studies (Murthy et al. 2003a,b), treatment of smooth muscle cells with CCh for 10 min caused a significant increase in Rho kinase activity in a concentration‐dependent fashion with an EC_50_ of 5 × 10^−9^ ± 1 × 10^−9^ mol/L (Fig. 6A). The concentration‐dependent curve was shifted to the right with EC_50_ values of 2.5 × 10^−8^ ± 4 × 10^−9^ mol/L and 9.8 × 10^−8^ ± 1.5 × 10^−8^ mol/L, respectively, by pretreatment of cells with l‐cysteine (10 mmol/L) or NaHS (1 mmol/L) for 10 min, suggesting inhibition of Rho kinase activity by H_2_S (Fig. 6A).

Pretreatment of cells with increasing concentrations of l‐cysteine or NaHS for 10 min caused inhibition of CCh‐stimulated Rho kinase activity and the inhibitory effect is concentration dependent with an EC_50_ of 5.6 × 10^−5^ ± 1.2 × 10^−5^ mol/L for NaHS and 9.9 × 10^−5^ ± 2.4 × 10^−5^ mol/L for l‐cysteine (Fig. 6B). Maximal inhibition was 89 ± 9% with NaHS (10 mmol/L) and 73 ± 7% with l‐cysteine (10 mmol/L). These results suggest that endogenous and exogenous H_2_S inhibits Rho kinase activity in colonic muscle cells. The kinase assay was specific for Rho kinase. CCh (1 μmol/L)‐stimulated Rho kinase activity was blocked by the addition of Rho kinase inhibitor Y27632 (10 μmol/L) (26,345 ± 3245 cpm/mg protein vs. 3214 ± 452 cpm/mg protein in the presence of Y27632, *n* = 5).

### Inhibition of Rho kinase activity by l‐cysteine is mediated via CSE/H_2_S

**Figure 7:**
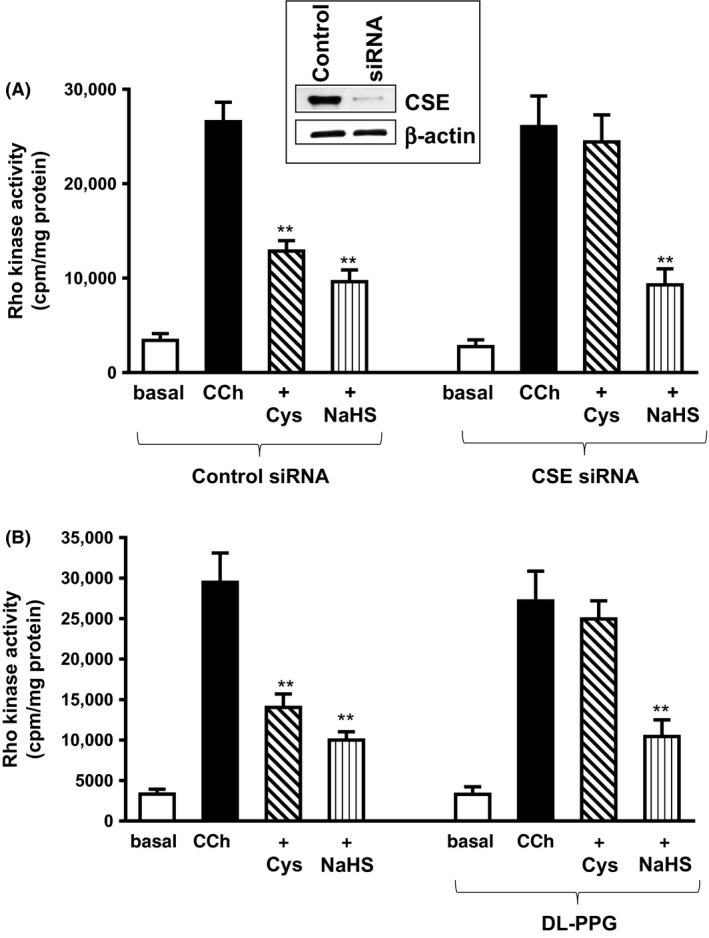
Inhibition of carbachol‐induced Rho kinase activity by l‐cysteine via activation of cystathionine‐γ‐lyase (CSE). (A) Cultured rabbit colonic muscle cells were transfected with control siRNA or CSE‐specific siRNA for 48 h. Cells were treated with carbachol (CCh; 1 μmol/L) for 10 min in the presence or absence of l‐cysteine (10 mmol/L) or NaHS (1 mmol/L) pretreatment for 10 min. Rho kinase activity was measured by immunokinase assay using [^32^P]ATP. Results are expressed as cpm/mg protein. Values are mean ± SE of 4–6 experiments. **P < 0.001, significant inhibition of CCh‐stimulated Rho kinase activity. Expression of CSE in cells transfected with control siRNA or CSE siRNA was analyzed by western blot (inset). (B) Dispersed muscle cells from rabbit colon were treated with CCh (1 μmol/L) for 10 min in the presence or absence of l‐cysteine (10 mmol/L) or NaHS (1 mmol/L) pretreatment for 10 min. In some experiments, cells were pretreated with CSE inhibitor, DL‐PPG (1 mmol/L) for 10 min before the addition of l‐cysteine (10 mmol/L) or NaHS (1 mmol/L) for another 10 min. Rho kinase activity was measured by immunokinase assay using [^32^P]ATP. Results are expressed as cpm/mg protein. Values are mean ± SEM of 4–6 experiments. **P < 0.001, significant inhibition of CCh‐stimulated Rho kinase activity.

The involvement of endogenous H_2_S generation by l‐cysteine to inhibit Rho kinase activity was tested by two methods: (1) by transfection with CSE‐specific siRNA in cultured muscle cells, and (2) by treatment with a selective CSE inhibitor, DL‐PPG in dispersed muscle cells. CCh induced a significant increase in Rho kinase activity in cultured muscle cells (28,819 ± 2312 cpm/mg protein; *P* < 0.001, *n* = 5) that is similar to the increase in dispersed muscle cells. Pretreatment of cells with l‐cysteine (10 mmol/L) for 10 min significantly inhibited CCh‐stimulated Rho kinase activity (63 ± 8% inhibition) in cells transfected with control siRNA and the inhibition was blocked in cells transfected with CSE‐specific siRNA (7 ± 1% inhibition, NS) (Fig. 7A), implying activation of CSE by l‐cysteine. Pretreatment of cells with NaHS (1 mmol/L) for 10 min also inhibited CCh‐stimulated Rho kinase activity in cells transfected with control siRNA (66 ± 7% inhibition) and the inhibition, however, was not affected in cells transfected with CSE‐specific siRNA (60 ± 7% inhibition).

Similar studies were performed in cells pretreated with DL‐PPG (1 mmol/L) for 10 min before treatment with l‐cysteine or NaHS in dispersed muscle cells. Pretreatment of cells with l‐cysteine (10 mmol/L) for 10 min significantly inhibited CCh‐stimulated Rho kinase activity (53 ± 7% inhibition) and the inhibition was blocked by DL‐PPG (1 mmol/L) (11 ± 5% inhibition, NS) (Fig. 7B). Pretreatment of cells with NaHS (1 mmol/L) for 10 min also significantly inhibited CCh‐stimulated Rho kinase activity in control cells (64 ± 8% inhibition) and the inhibition, however, was not affected by DL‐PPG (65 ± 3% inhibition) (Fig. 7B). These results provide evidence for the involvement of CSE, via generation of H_2_S, in the inhibition of Rho kinase activity by l‐cysteine.

**Figure 8:**
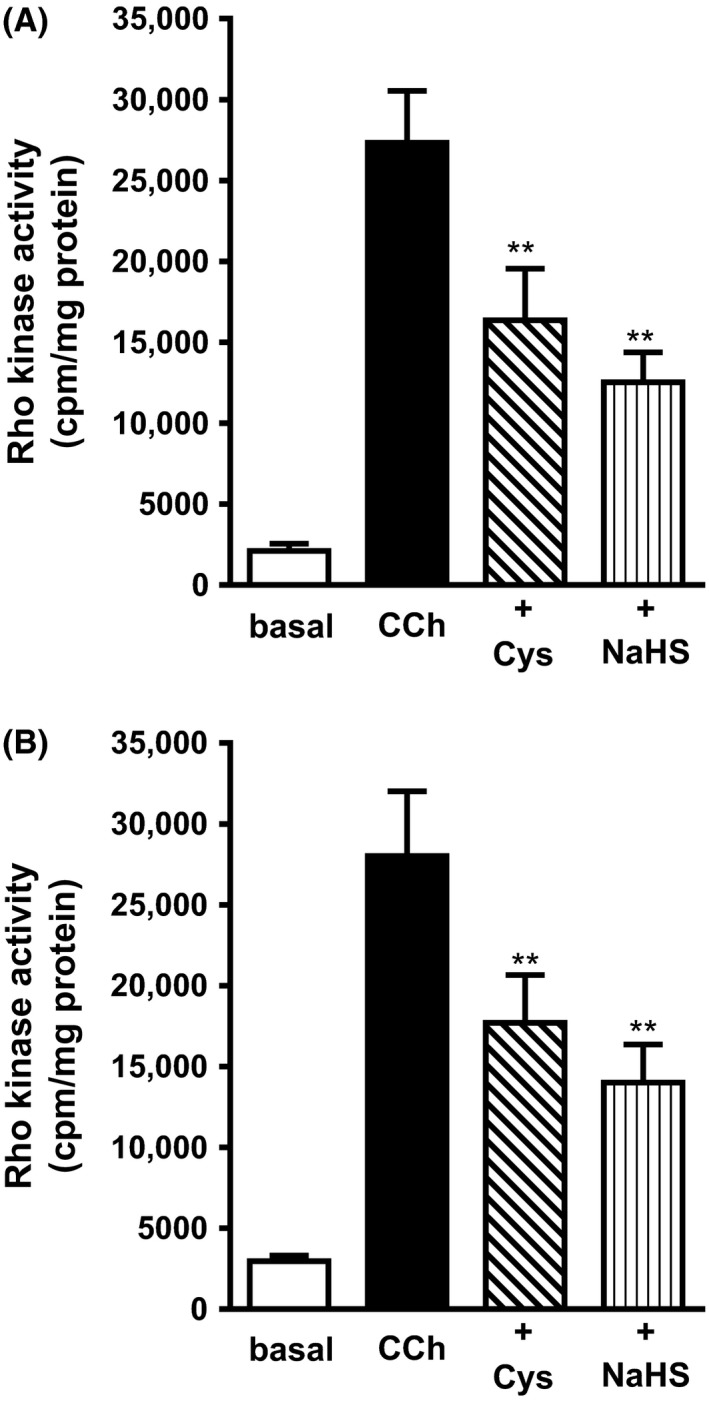
Inhibition of carbachol‐induced Rho kinase activity by l‐cysteine and NaHS in colonic muscle cells. Muscle cells isolated from mouse (upper panel, A) or human (lower panel, B) colon were pretreated with l‐cysteine (10 mmol/L) or NaHS (1 mmol/L) for 10 min and then treated with CCh for 10 min. Rho kinase activity was measured by immunokinase assay using [^32^P]ATP. Results are expressed as cpm/mg protein. Values are mean ± SEM of 4–6 experiments. **P < 0.01 significant inhibition of CCh‐stimulated Rho kinase activity.

Pretreatment of cells with l‐cysteine (10 mmol/L) or NaHS (1 mmol/L) for 10 min also inhibited Rho kinase activity in response to CCh in mouse and human. In mouse colonic muscle cells, inhibition by l‐cysteine was 43 ± 6% and inhibition by NaHS was 58 ± 5% (Fig. 8A). In human colonic muscle cells, inhibition by l‐cysteine was 40 ± 4% and inhibition by NaHS was 54 ± 6% inhibition (Fig. 8B).

## Discussion

Recent studies have demonstrated that H_2_S, like nitric oxide (NO) and carbon monoxide (CO), is justified to be defined as a physiological transmitter (Linden et al. 2010; Wang 2012). Like NO and CO, H_2_S is a small gaseous and freely permeable molecule. The physiological importance of H_2_S has been underscored by its significance in the regulation of several functions including GI functions (Distrutti et al. 2006; Linden et al. 2008; Hennig and Diener 2009; Xu et al. 2009; Wallace 2010; Strege et al. 2011; Sha et al. 2014; Nalli et al. 2015). H_2_S is endogenously synthesized via CSE, CBS, and 3‐mercaptopyruvate sulfurtransferase (Wang 2012). Of these three enzymes, CSE and CBS have been well studied. Both enzymes are dependent on pyridoxal‐5’‐phosphate and use l‐cysteine as a substrate to produce H_2_S (Wang 2012; Farrugia and Szurszewski 2014). Although the pattern of expression of CSE and CBS in various tissues is largely known, the regulatory mechanisms for expression and activities are not clear. Using exogenous H_2_S donors (e.g., NaHS) or l‐cysteine (substrate of CSE), pharmacological inhibitors of enzymes that generate H_2_S (e.g., DL‐PPG) and molecular approaches (siRNA, and CSE/CBS null mice), several functions of H_2_S have been demonstrated in the cardiovascular system, central nervous system, GI tract, and energy metabolism (Abe and Kimura 1996; Yang et al. 2008; Linden et al. 2010; Wang 2012). Studies from the ablation of CSE or downregulation of CSE highlight the importance of endogenous H_2_S in the regulation of smooth muscle relaxation (Zhao et al. 2001; Yang et al. 2008). One of the most studied targets of H_2_S is the K_ATP_ channel. The effect of H_2_S on vascular muscle is due to the activation of K_ATP_ channels, whereas inhibition of K_ATP_ channels with glibenclamide blocked the effect of H_2_S (Zhao et al. 2001; Zhao and Wang 2002; Tang et al. 2005; Mustafa et al. 2011; Wang 2012). The relaxant effect of NaHS in mouse aorta and bronchial rings, however, was not affected by glibenclamide (Kubo et al. 2007a,b). An excitatory action of H_2_S on bladder contraction involving capsaicin‐sensitive nerves was reported (Pattacchini et al. 2004).

Although H_2_S is known to cause inhibition of contraction both in vivo and in vitro in the GI tract, the underlying mechanism of action of H_2_S is not clear. Targets such as voltage‐dependent Ca^2+^ channels and Ca^2+^‐dependent K^+^ channels are implicated in mediating the effect of H_2_S in rat colonic smooth muscle cells (Quan et al. 2015). Inhibition of contraction mediated by H_2_S in rabbit and guinea pig ileum, and in rat longitudinal muscle was not affected by the K_ATP_ channel blocker, glibenclamide (Teague et al. 2002; Nagao et al. 2011). NaHS‐induced inhibition of spontaneous contraction in isolated segments of mouse colon and jejunum was unaffected by tetrodotoxin, capsaicin, and *N*‐nitro‐l‐arginine, but reduced by apamin (Gallego et al. 2008). Tetrodotoxin also had no effect on the relaxation in the distal colon of mouse and human suggesting a direct effect on smooth muscle (Gade et al. 2013; Martinez‐Cutillasa et al. 2015; Quan et al. 2015). These studies suggest that the underlying mechanism of action of H_2_S to inhibit contraction varies with the tissue and species. Increase in spontaneous contraction in the presence of CSE inhibitors suggests a role for endogenous production of H_2_S via CSE in the regulation of GI motility (Martinez‐Cutillasa et al. 2015). In the GI tract, the loci of generation and action of H_2_S include epithelial cells, enteric neurons, ICCs, and smooth muscle cells.

In the present study, we investigated the expression of H_2_S synthesizing enzymes and identified the mechanisms underlying the inhibitory effects of H_2_S in the smooth muscle from the colon of rabbit, mouse, and human. Our findings include: (1) selective expression of CSE in smooth muscle cells of rabbit, mouse, and human, where it is responsible for H_2_S production; (2) inhibition of CCh‐induced contraction by l‐cysteine and NaHS in muscle strips and dispersed smooth muscle cells of rabbit and blockade of inhibition by a selective inhibitor of CSE (DL‐PPG), but not by a selective inhibitor of K_ATP_ channels (glibenclamide); (3) S‐sulfhydration of RhoA by NaHS and attenuation of CCh‐induced RhoA and Rho kinase activities and muscle contraction by both l‐cysteine and NaHS; (4) blockade of l‐cysteine‐induced inhibition by DL‐PPG or CSE siRNA; and (5) inhibition of Rho kinase activity and muscle contraction by l‐cysteine and NaHS in colonic smooth muscle cells from mouse and human.

Expression of CSE and CBS are tissue and species specific. In rat colon, mucosal cells, myenteric neurons, and smooth muscle cells were immunoreactive for CBS and CSE (Quan et al. 2015). In rat jejunum, myenteric neurons, but not smooth muscle were immunoreactive for CBS and CSE (Kasparek et al. 2012). In mouse colon, mucosal cells were immunoreactive for CBS and CSE, whereas myenteric neurons were immunoreactive for only CSE (Linden et al. 2008). In the colon of human and guinea pig, submucosal cells and myenteric neurons were immunoreactive for CBS and CSE, whereas only myenteric interstitial cells of Cajal were immunoreactive for CSE (Schicho et al. 2006). Expression of H_2_S synthesizing enzymes also varies with the different regions of the GI tract. Expression of CSE is more abundant in the proximal regions (stomach, duodenum, and jejunum), compared to distal regions (ileum and colon) of rat GI tract. In contrast, expression of CBS was low in proximal regions (duodenum and jejunum) and high in the distal regions (ileum and colon) (Martin et al. 2010). Our studies using cultured muscle cells devoid of enteric neurons and ICCs clearly demonstrated selective expression of CSE in smooth muscle cells. This agrees with the previous reports that CBS expression is abundant in central nervous system, and rare in a peripheral system (Bao et al. 1998; Linden et al. 2010; Wang 2012).

l‐Cysteine and/or NaHS were commonly used in vivo and in vitro, in whole organ and nerve‐muscle preparation, to examine the physiological significance of H_2_S in the regulation of GI motility. Although H_2_S inhibits contractions, as discussed above, there are considerable differences in the mechanism of action. Interpretation of results was also confounded by the multiple cells types present in the tissue preparation used in these experiments. Our studies showed that both endogenous H_2_S, generated via CSE, and exogenous H_2_S inhibit sustained contraction in muscle cells, suggesting a direct effect of both endogenous and exogenous H_2_S on smooth muscle cells. The concentrations of NaHS and l‐cysteine used in our studies are similar to those used in other studies to induce a response (Distrutti et al. 2006; Gallego et al. 2008; Dhaese and Lefebvre 2009; Gil et al. 2011; Kasparek et al. 2012; Nagao et al. 2012). These high concentrations reflect concentrations of H_2_S closer to the target site but not a global tissue concentration due to variations in the metabolism of H_2_S in different cellular compartments (Levitt et al. 2009; Wang 2012). The recovery of contractile activity after washout of the initial application of l‐cysteine and NaHS was rapid and complete, suggesting that the concentrations used in the study are not toxic. In most of the in vitro studies, a significant effect was produced with a tissue bath H_2_S concentration of high μmol/L (100 μmol/L) or mmol/L (d'Emmanuele diVilla Bianca et al. 2009; Bucci et al. 2012). These concentrations have been considered to be physiologic, as several reports suggest that the tissue H_2_S concentration normally ranges from high μM to low mM range (30 μmol/L to >100 μmol/L) (Yang et al. 2012). The free H_2_S concentrations in GI smooth muscle and the significance of H_2_S at low μM concentration as a messenger molecule are yet to be determined. Our studies also showed that the effect of H_2_S is not dependent on the activation of K_ATP_ channels, but dependent on the S‐sulfhydration of RhoA.

In the present study, we have identified RhoA as S‐sulfhydration target of H_2_S in mediating the inhibitory effect on muscle contraction. Like S‐nitrosylation by nitric oxide, S‐sulfhydration by H_2_S appears to be the common post‐translational mechanism to alter the function of proteins (Mustafa et al. 2009; Wang 2012). S‐sulfhydration of several proteins including receptors, ion channels, and enzymes have been described in previous studies (Mustafa et al. 2009; Wang 2012). Initially, it was suggested that S‐sulfhydration always results in the increase of protein activity; however, the inhibitory effect of sulfhydration was also demonstrated (Mustafa et al. 2009; Wang 2012). Previous studies in gastric muscle strips demonstrate regulation of MLC phosphatase activity by H_2_S (Dhaese and Lefebvre 2009). In GI smooth muscle, activation of RhoA by contractile agonists causes sustained contraction via phosphorylation of the regulatory subunit of MLCP (MYPT1) and inhibition of MLC phosphatase leading to an increase in MLC_20_ phosphorylation and muscle contraction (Murthy et al. 2003a; Murthy 2006). H_2_S exerts an inhibitory effect on RhoA activity causing attenuation of Rho kinase activity and disinhibition of Rho kinase‐mediated MLCP activity, augmentation of MLC_20_ dephosphorylation, and inhibition of sustained contraction. Blockade of l‐cysteine effect by DL‐PPG in dispersed muscle cells and CSE siRNA in cultured muscle cells provides evidence for the involvement of CSE activation.

In summary, our studies demonstrated the selective expression of CSE in colonic smooth muscle cells and identified the molecular mechanism by which H_2_S inhibits muscle contraction via S‐sulfhydration of RhoA and inhibition of RhoA and Rho kinase activities.

## Author Contributions

K. S. M., A. N., and H. W. participated in research design; A. N., H. W., S. B., B. B., and K. S. M. conducted experiments; K. S. M. and A. N. performed data analysis; K. S. M., A. N., and S. B. wrote or contributed to the writing of the manuscript. None contributed to new reagents or analytical tools.

## Disclosure

None declared.
